# The Guest Hospital, Dudley

**Published:** 1903-09-12

**Authors:** 


### Editor's Letter-Box.

**[Our Correspondents are reminded that prolixity is a great bar to publication, and that brevity of style and conciseness of statement greatly facilitate early insertion.]**

### THE GUEST HOSPITAL, DUDLEY.

We have received a letter from the late resident medical officers of the Guest Hospital, Dudley, Messrs. E. C. Hadley, F.R.C.S.E., and C. Rolleston, M.B., B.Ch.Oxon., in which they ask our opinion on a question of hospital discipline. We have not space for their entire letter, but briefly the case is as follows:—A patient was admitted to the Guest Hospital with a fractured leg, due to a severe crush. Sloughing of the skin and soft tissues at the site of the crush ensued, and amputation became necessary and was performed after some delay, caused by the patient's refusal to give his consent. Throughout the time previous to the performance of amputation the patient had been extremely insubordinate, refusing to keep his splints on, using obscene language, and indecently exposing himself to the nurses. After repeated warnings he was forcibly expelled from the hospital, with the consent of the hospital secretary and of the surgeon in charge of the case. He was subsequently readmitted by the surgeon in charge, to whom he had apologised and promised to behave properly in the future. After the patient was readmitted the resident medical officers found that it was impossible to maintain discipline in the ward; they consequently refused to treat the patient and immediately reported the matter to the visitors of the month. The Board at its next meeting had the matter put before them and fully approved of the expulsion of the patient. The resident medical officers then expressed their intention to immediately resign unless the patient were removed from the hospital. Two alternative propositions were made to them, one of which was that they should continue to treat the patient if he would apologise, and the other was that their resignations should be accepted and that the patient in question should be at once discharged from the hospital, in order that the resident medical officers might be able to continue their duties for three months, which was the necessary notice of resignation of the senior post. They accepted the latter course except that they would not agree to the usual notice under the circumstances, the patients being then so extremely unruly, and the nurses saying that they felt that they had no safeguard if the R.M.O. were so treated. The resident medical officers are now anxious to know whether we approve of their attitude, and whether it would be unprofessional conduct for another registered practitioner to accept their late posts, on such terms.

[We are not in a position to decide upon the merits of the case as we have only heard the statements of one party, but judging from those statements it does appear that some want of regard was displayed by the hospital authorities towards the resident medical officers, the nurses and the patients who were in proximity to the insubordinate patient; at the same time we cannot help feeling that the resident medical officers would have done well to extend their forbearance so far as accepting the second alternative which was offered to them, and continuing to serve the necessary three months after the expulsion of the patient. With regard to the last question raised we cannot see that it would be in any way unprofessional conduct for another registered practitioner to accept one of the vacant posts on the same terms as it was held previously.

Ed. The Hospital.]

